# Editorial: Microencapsulation for Biomedical Applications

**DOI:** 10.3389/fbioe.2022.891981

**Published:** 2022-03-25

**Authors:** Aurelio Salerno, Filippo Causa, Concetta Di Natale, Concepción Domingo, Raffaele Vecchione

**Affiliations:** ^1^ Independent Researcher, Barcelona, Spain; ^2^ Interdisciplinary Research Centre on Biomaterials (CRIB), Università degli Studi di Napoli “Federico II”, Naples, Italy; ^3^ Dipartimento di Ingegneria Chimica dei Materiali e della Produzione Industriale (DICMAPI), University “Federico II”, Naples, Italy; ^4^ Institut de Ciència de Materials de Barcelona (ICMAB-CSIC), Barcelona, Spain; ^5^ Center for Advanced Biomaterials for Health Care (iit@CRIB), Istituto Italiano di Tecnologia, Naples, Italy

**Keywords:** drug delievery systems, microencapculation, tissue engineering, emulsion, scaffold, nanoparticles, nanotubes, cell encapsulation

**FIGURE 1:**
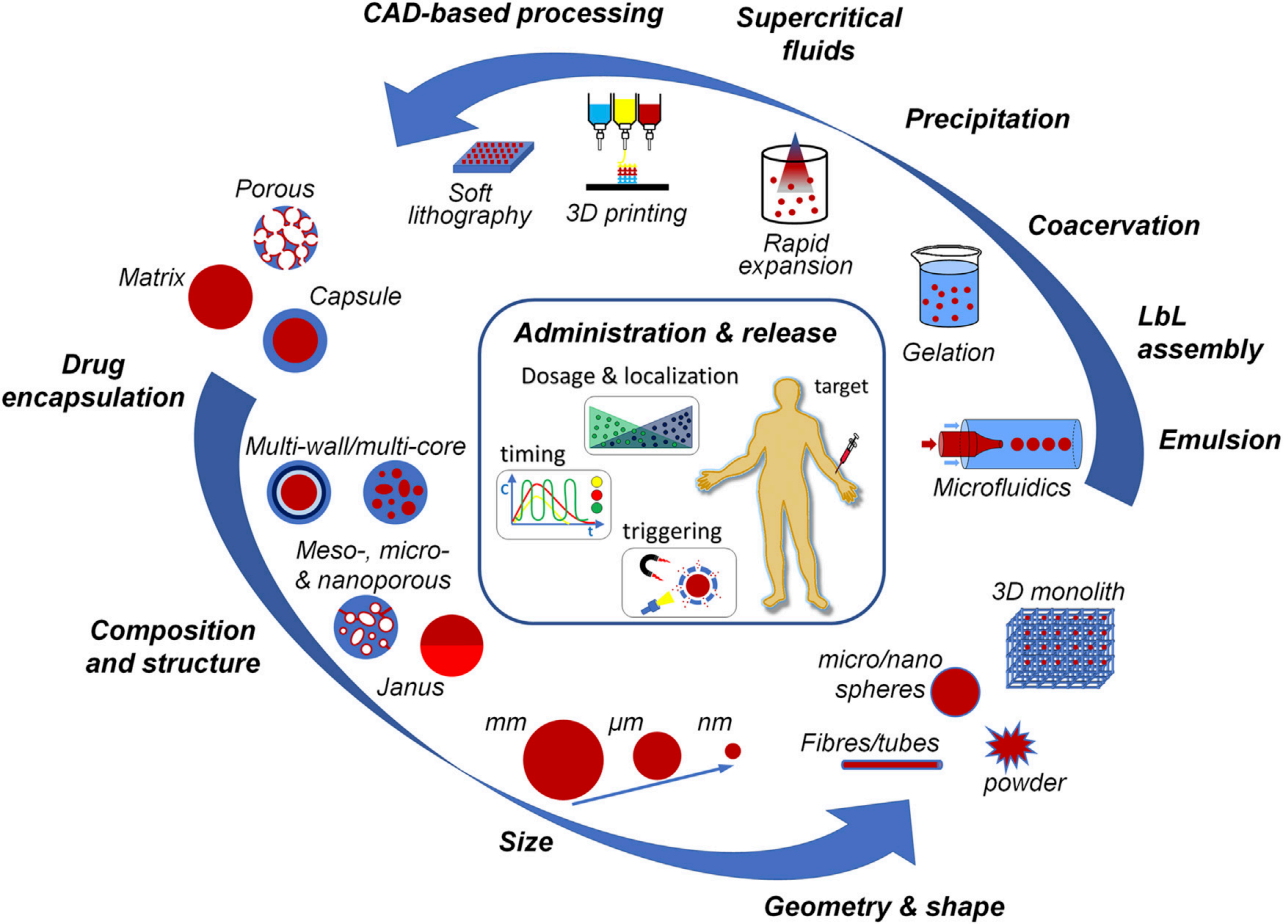
Picture highlighting the main properties of drug delivery carriers and some of the most advanced processing techniques for carriers manufacturing. The drug can be encapsulated within the polymeric matrix (particles), loaded in the core (single and multi-core capsules) or adsorbed onto the inner pore surfaces. The control of drug release is achievable by tuning carriers’ composition, structure, size and shape and by the optimization of the fabrication technique and processing conditions.

The systemic administration of drugs, such as chemotherapeutics, probiotics and anti-inflammatories, is known to be frequently associated to biomolecules short half-life, poor stability and side effects potentially harmful to functional tissues and organs. Advanced drug therapies require patient customization and targeting of drug formulation and dosage to warrant treatment efficacy and reduce possible undesired secondary effects. Nano- and microencapsulation strategies are generally defined as a set of technologies that allow to entrap active ingredients, namely small solid particles, liquid droplets or a gas, using a surrounding material (Silva and Meireles, 2014). These strategies may allow overcoming previous limitations as the encapsulating material protects the drugs, while their delivery can be tailored depending on the specific application by the careful modulation of carrier composition, size and architectural features. A variety of methods, including microfluidic emulsion, coacervation, antisolvent precipitation and soft lithography, have been used to produce drug delivery systems for precision medicine (Wang et al., 2006; Canelas et al., 2009; Liu et al., 2017). Overall, these methods offer control over basic parameters such as material-to-drug composition, carrier size, porosity and shape (Figure 1). In this vast and complex panorama, the aim of this Research Topic is to highlight and illustrate important knowledge in the field of micro- and nanoencapsulation and drug delivery systems as shared by experts in these research fields. Most notably, the nine articles collected in this issue, composed of five research articles and four reviews by different countries’ teams, are of interest to researchers looking for current drug delivery advances in biomedicine and biotechnology, as described following.

Two articles of this collection are specifically focused on the application of hydrogels on cell microencapsulation for tissue engineering (TE) purposes. TE offers great potential for restoring individual tissues or organs using patient’s stem cells incorporated within porous scaffolds or hydrogels (Khademhosseini et al., 2009). However, low new tissue survival, poor engraftment and a lack of site-specificity are major drawbacks. Natural polymers have gained much interest in the construction of extracellular matrix (ECM) analogues for stem cells. Cell-laden hydrogel microspheres with uniform size show great potential for tissue repair and drug screening applications. The article by Lee et al. reviewed the application of polysaccharide-based hydrogels for stem cells microencapsulation in TE. In particular, the work showed an updated vision of microencapsulation techniques, which include emulsion, lithography, microfluidics and bioprinting. Furthermore, current progress in clinical translation of stem-cell encapsulated polysaccharide hydrogels for cell delivery and disease modelling (drug testing and discovery) were discussed with special emphasis on musculoskeletal, nervous, cardiac and cancerous tissues applications. In the article by Zhang et al., a simple one-step approach for producing and purifying hydrogel microspheres with an easily assembled microfluidic device was described. Droplets were generated and solidified within a fluidic device and then demulsification and purification from oil was obtained by the simple evaporation of the oil at 37°C. This step allowed gelled microspheres to be released directly into the cell culture media, ready to be tested for cell culture experiments. HCT116 and U87 tumour cells were successfully encapsulated within monodisperse gelatin methacryloyl microspheres ensuring proper cell viability. The U87-encapsulated microspheres were also used to growth tumour spheroids up to 14 days, finally suggesting the possible translational technology of the developed approach. As previously explained, drug delivery scaffolds are essential elements of TE strategies for the repair and regeneration of damaged tissues and organs. Indeed, scaffolds must endow with arrays of biological signals, with adequate dose and timescale, to regulate cellular adhesion, migration and ECM biosynthesis in three dimensions (Salerno et al., 2019). Therefore, scaffolds fabrication requires the concomitant processing of biomaterials, cells and biomolecules to reproduce the cell and ECM composition and architecture of native tissues. The use of computer-aided design (CAD) and manufacturing of drug delivery scaffolds was reviewed by the work of Salerno and Netti. The article highlights some of the most recent advancement of CAD-based strategies to engineering passive and stimuli-responsive drug delivery scaffolds for TE and cancer precision medicine. Furthermore, authors’ perspective about the possible integration of CAD techniques with microfluidics and soft lithography was reported for enhancing scaffold bioactivation features.

A valuable application of microencapsulation techniques is to protect probiotics from harmful gastrointestinal tract (GI) environmental factors, such as high acidity and low pH levels, bile salts, and oxidation conditions. This issue was addressed by the work of Di Natale et al., that encapsulated the *L. paracasei CBA L74* bacteria in sodium alginate microspheres by the water-in-oil emulsion technique to protect it in GI and to enhance its viability and beneficial effects. The optimal microencapsulation conditions were obtained by using a micro-rheological analysis as it allowed to understand the relationship between emulsion conditions and microsphere’s inner microstructure, which in turn can affect probiotic viability and release.

Nanocarriers are highly versatile and valuable systems for drugs delivery as they provide high specific surface together with small size, typically between 1 and 100 nm. Due to these features, nanocarriers may provide a long-term circulation period with the sustained release of drug, overcoming limitations related to the endosomal/lysosomal membrane transport (Chamundeeswari et al., 2019). Among the different forms of nanocarriers, nanofibers, nanoparticles and nanotubes, are the most investigated as they effectively found application in the biomedical field. In the article by Oseni et al., the chemotherapeutic andrographolide was encapsulated within polylactic-co-glycolic (PLGA) nanoparticles *via* emulsion solvent evaporation technique. The effect of polymer composition, polymer molecular weight, polymer-to-drug ratio, surfactant concentration and the organic solvent used on particles size and encapsulation efficiency was investigated. Nanoparticles formulated using 85:15 PLGA and ethyl acetate as the organic solvent provided the best particle size, drug loading and release towards the inhibition of proliferation of metastatic breast cancer cells. In another study, Sandoval and Tobias used fullerenes to “cork” the open tips of multiwalled carbon nanotubes (MWCNTs), and as promoting species for the on-demand release of the inorganic material filled within the nanotubes’ cavities. In particular, fullerenes avoided the release of the encapsulated payloads during samples washing as well as may enable to trigger the release of guest structures from the MWCNTs cavities once dissolved in appropriate solvents. Most notably, the authors demonstrated that MWCNTs can be loaded with chosen bioactive compound before thermal treatment, therefore allowing the loading of heat labile biomolecules within MWCNTs porosity. The application of micro- and nanoencapsulation to the antioxidant, antimicrobial, and therapeutic oregano essential oil (OEO) has been reviewed by the work of Pontes-Quaero et al. In fact, encapsulation is necessary to increase OEO stability and bioactivity and to decrease its volatility. Hence, different drug delivery systems, mainly lipids and cyclodextrins, were discussed respect to scientific literature, in order to find the best candidates for OEO encapsulation. Among different natural polymers, bacterial cellulose (BC) is a highly pure form of cellulose produced by bacteria that can be chemical-physically modulated and optimized to encapsulate and deliver several drugs. The work by Di Natale et al. described the preparation of antioxidant and anti-inflammatory nanofibrous patches by loading CoenzymeQ10 (Co-Q10) nanoemulsions within the porous structure of BC. To this purpose, BC layers were incubated at different time points with a positively-charged oil/water nanoemulsion, previously loaded with Co-Q10, and the efficacy of release was studied at different time points.

The last article presented in this Research Topic Editorial (Duong et al.) rely on the use of porous aerogel particles for pulmonary drug delivery. Aerogels, the lightest processed solid materials on Earth, have enormous composition versatility, modularity, and feasibility of industrial scale manufacturing, facts which are behind the fast emergence of aerogels in the drug delivery field. Particularly, the physical properties of the aerogels appear to be very advantageous for mucosal administration routes, such as pulmonary, nasal, or transdermal. This article in fact gives important insights regarding the use of low-density aerogels for pulmonary administration, both for local treatment of lung diseases and for the systemic delivery (transpulmonary) of labile biopharmaceuticals, including those for gene therapy and vaccination.

In conclusion, all of the articles collected in this Research Topic are proof of the increasing knowledge and importance of micro- and nanoencapsulation for drugs and bioactive molecules delivery. Thanks to the advancement on drug discovery and micro- and nanomaterials processing, it is now possible to design and engineering multifunctional tailor-made drug delivery systems for TE, biotechnology and health care.
